# Understanding the Impact of the Three-Dimensional Junction Thickness of Electrospun Bipolar Membranes on Electrochemical Performance

**DOI:** 10.1021/acsapm.2c02182

**Published:** 2023-04-04

**Authors:** Emad Al-Dhubhani, Jan W. Post, Marat Duisembiyev, Michele Tedesco, Michel Saakes

**Affiliations:** † Wetsus, European Centre of Excellence for Sustainable Water Technology, Oostergoweg 9, 8911 MA Leeuwarden, The Netherlands; ‡ L.N. Gumilyov Eurasian National University, Satpayev str. 2, 010008 Astana, Repulic of Kazakhstan; § Membrane Science and Technology, University of Twente, P.O. Box 217, 7500 AE Enschede, The Netherlands

**Keywords:** bipolar membrane, 3D junction thickness, electrospinning, water dissociation, reverse bias, forward bias

## Abstract

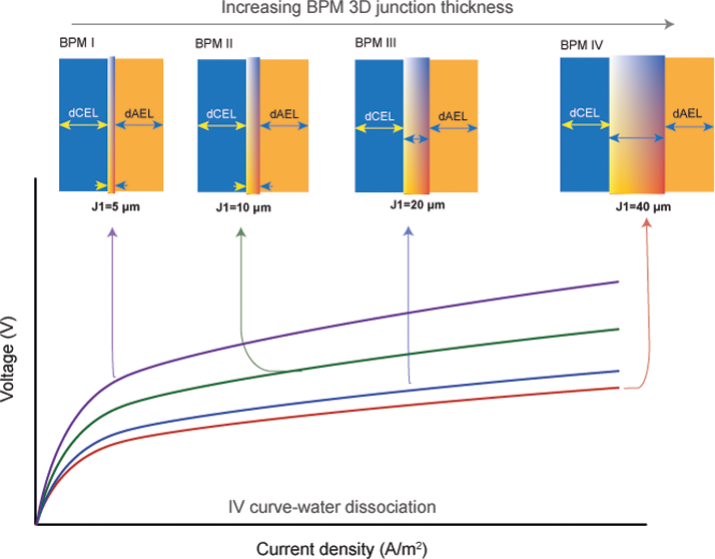

The use of electrospun bipolar membranes (BPMs) with an interfacial three-dimensional (3D) junction of entangled nano-/microfibers has been recently proposed as a promising fabrication strategy to develop high-performance BPMs. In these BPMs, the morphology and physical properties of the 3D junction are of utmost importance to maximize the membrane performance. However, a full understanding of the impact of the junction thickness on the membrane performance is still lacking. In this study, we have developed bipolar membranes with the same composition, only varying the 3D junction thicknesses, by regulating the electrospinning time used to deposit the nano-/microfibers at the junction. In total, four BPMs with 3D junction thicknesses of ∼4, 8, 17, and 35 μm were produced to examine the influence of the junction thickness on the membrane performance. Current–voltage curves for water dissociation of BPMs exhibited lower voltages for BPMs with thicker 3D junctions, as a result of a three-dimensional increase in the interfacial contact area between cation- and anion-exchange fibers and thus a larger water dissociation reaction area. Indeed, increasing the BPM thickness from 4 to 35 μm lowered the BPM water dissociation overpotential by 32%, with a current efficiency toward HCl/NaOH generation higher than 90%. Finally, comparing BPM performance during the water association operation revealed a substantial reduction in the voltage from levels of its supplied open circuit voltage (OCV), owing to excessive hydroxide ion (OH^–^) and proton (H^+^) leakage through the relevant layers. Overall, this work provides insights into the role of the junction thickness on electrospun BPM performance as a crucial step toward the development of membranes with optimal entangled junctions.

## 1 Introduction

Bipolar membranes (BPMs) have been recognized in the past few years as promising tools to unlock several applications toward a more sustainable and circular use of resources. BPMs are polymeric membranes with the ability to catalyze the dissociation of water into acid and base at the junction between the cation- and anion-exchange layers. Moreover, it is also possible to use bipolar membranes in the opposite way (forward bias) to neutralize the acid and base at opposite sides of the BPM to form water.

The unique properties of bipolar membranes led to the invention of many electrochemical-based processes, such as organic acid and base production, CO_2_ capture,^1−4^ adjustment of the pH of water and juices,^5,6^ protein separation,^7,8^ and ammonia or acid recovery from wastewaters.^9,10^ Recent applications involve the use of BPMs in fuel cells to facilitate the optimal pH regulation at each electrode.^11,12^ With the use of bipolar membrane electrodialysis (BMED), oxygen evolution and hydrogen evolution reactions (OER and HER) are promoted for faster kinetics using more abundant catalysts such as nickel.^13−16^

BPM characteristics and performance were investigated in relation to the thicknesses of the monopolar cation- and anion-exchange layers of the laminated or cast BPMs.^17^ Asymmetrical homogeneous and heterogeneous BPMs of different anion- and cation-layer thicknesses were fabricated in previous studies.^18,19^ These studies demonstrated the influence of the BPM ion-exchange layer thickness on the purity of the produced acid and base. Wilhelm et al. reported that the transport of salt ions in a BPM has an inversely proportional relationship with the fixed current density and the thickness of ion-exchange layers of the BPM.^18,20^

Recently, electrospinning has been utilized to fabricate BPMs in a unique fabrication route where cationic and anionic nanofibers are entangled at the interface to form a three-dimensional (3D) junction.^21−24^ It has been proven that such a method can create a single-film BPM with excellent adhesion and superior performance compared to the conventionally laminated BPM made of two (or three) single layers.^21,22,24^

Electrospun BPMs mostly consist of three identifiable layers: the cation-exchange layer, the anion-exchange layer, and the (3D) junction, wherein the junction is composed of entangled cation- and anion-exchange fibers. Moreover, the junction layer typically includes a water dissociation catalyst, such as metal (hydr)oxides^25^ (e.g., Al (OH)_3_, SiO_2_,^21^ etc.), polymers like poly (4-vinyl pyridine) (P4VP),^24^ electronically conducting polymers like polyaniline (PANI),^26^ and graphene oxide (GO)^22,23^ introduced during the fabrication. The thickness of this 3D junction is crucial for the performance of the BPM as it operates at the interfacial/water dissociation region, where most of the water dissociation is hypothesized to occur.

Such BPM-3D junctions have been reported with various thicknesses, although made of various compositions of ion-exchange polymers and catalysts. For example, some of the 3D junction thicknesses presented in the literature are 3–5,^23^ 10,^21^ and 20 μm,^24^ accounting for 5–8, 22, and 25% of the total BPM thicknesses, respectively.

Despite the above-mentioned studies, a clear understanding of the effect of the junction thickness on BPM performance is still lacking, especially because a direct comparison between previous studies is rather difficult due to the different polymeric compositions and working conditions. Therefore, isolating the effect of junction thickness under controlled operating conditions and chemistry is yet lacking in the literature. Kole et al. previously investigated the variation of the BPM interfacial area through the method of soft lithography, where micropatterned BPMs had an enlargement of 2.28× of the interfacial active area. That study focused on changing the interfacial area of the originally planar interface. However, in this work, the interfacial active area of the 3D junction is varied for an electrospun BPM through the method of electrospinning.^27^ In this study, several symmetrical BPMs with 3D junctions are fabricated with different junction thicknesses, keeping the polymeric composition of each layer and the controlled operating conditions identical. By changing the 3D junction thickness, the total anion/cation membrane contact surface area is altered such that the influence of the 3D junction thickness on the BPM performance is studied while keeping all other parameters constant. The aim of this study is to investigate the effect of changing the BPM 3D junction thickness on the performance of the bipolar membrane for water dissociation. It is important to emphasize that the total contact specific area changes together with the junction thickness. For all fabricated BPMs, we investigated the performance in terms of the current efficiency (as a function of the junction thickness) as well as the purity of the produced acid (HCl) and base (NaOH).

## 2 Materials and Methods

### 2.1 Membrane Fabrication via Electrospinning and Hot-Pressing

The methodology of fabricating the BPMs using the electrospinning/hot-pressing approach has been thoroughly reported in our previous study.^24^ The anion-exchange polymer FAA-3 (with an ion-exchange capacity, IEC, of 2 meq/g) was prepared by dissolving in dimethylacetamide (DMAc) at a weight concentration of 26 wt %. Poly(4-vinylpyrrolidone) (P4VP) was blended in the FAA-3 solution with a resulting percentage of 15 wt % in FAA-3. Cation-exchange polymer solutions were prepared by dissolving commercially provided SPEEK (with an ion-exchange capacity, IEC, of 1.9 meq/g) at 20 wt % in DMAc. Table 1 provides the main parameters of electrospinning used to fabricate the BPMs. Following the process of electrospinning, the electrospun BPM mat was converted into a dense layer by hot-pressing. For this, the mat was placed in between two PTFE sheets, which were, in turn, placed between two metal plates. The hot-pressing process was then conducted with a hot-pressing machine (P300S, VOGT, Labormaschinen GmbH, Germany) at 150 °C and 200 bars for 1 h.

**Table 1:** Electrospinning Parameters Followed for BPM Fabrication

	anion-exchange nanofibers	cation-exchange nanofibers
polymer	FAA-3	SPEEK
solvent	dimethylacetamide, DMAc	
substrate	polyethylene carbon black (PECB)	
working distance	100 mm	75 mm
temperature	30 °C	
drum rotation rate	200 rpm	
negative voltage (*V*_drum_)	–10 kV	
positive voltage (*V*_tip_)	+6.0 kV	+18 kV

**Figure 1:**
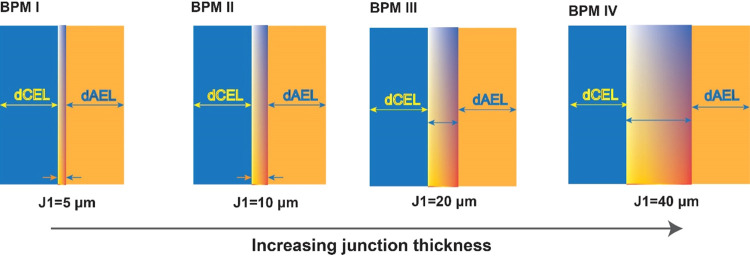
Illustration of different fabricated BPMs with varying 3D junction thicknesses. Note that the thicknesses of the CEL and AEL (*d*_CEL_ = 30 μm, *d*_AEL_ = 30 μm) are maintained constant for all membranes.

The summary of different bipolar membranes prepared by varying the junction thickness, targeting several 3D junction thicknesses, is presented in Table 2. This was achieved by changing the electrospinning time for depositing the polymer fibers to change the junction thickness. As illustrated in Figure 1, four thicknesses were targeted, namely, 5, 10, 20, and 40 μm, as estimated values, which were measured afterward using SEM-EDX analysis.

**Table 2:** Main Properties of the Fabricated BPMs and Electrospinning Operating Times

						time for dual electrospinning using two single needles		
ES-HP BPM	anion layer composition	cation layer composition	interface layer	junction thickness (targeted)	total BPM thickness	anion-exchange layer (h)	junction (h)	cation-exchange layer (h)
BPM I	FAA-3 and P4VP	SPEEK	entangled fibers of SPEEK and (FAA-3/P4VP) polymer blend	5	65	3	0.5	3
BPM II				10	70	3	1	3
BPM III	polymer content (85:15)			20	80	3	2	3
BPM IV				40	100	3	4	3

### 2.2 Scanning Electron Microscopy (SEM) Analysis and Elemental Mapping (EDX)

Cross-sectional imaging was conducted by scanning electron microscopy (SEM)–energy-dispersive X-ray (EDX) analysis (JEOL JSM-6480 LV). The cross-sectional images were used to examine post-treated electrospun membranes and provide the best estimation of the thickness of each layer. All BPMs were immersed in 2 M NaCl solution and dried in a vacuum oven at 50 °C overnight to remove all of the moisture content before starting the characterizations.

### 2.3 Specific Surface Analysis with Nitrogen Adsorption–Desorption

Electrospun samples for the fabricated BPM I, BPM II, BPM III, BPM IV, and 3D junction, with weights between 0.3 and 0.5 mg, were analyzed before the process of hot-pressing to evaluate their specific surface areas (in m^2^/g). Before performing the Brunauer–Emmett–Teller (BET) analysis, samples were degassed at 105 °C overnight (∼18 h) in a VacPrep 061 degasser (Micromeritics). The BET specific surface area (BET) and porosity were determined at standard temperature and pressure (77 K) using a BET analyzer Tristar II Plus (Micromeritics, US) and with nitrogen gas as the adsorptive gas.

### 2.4 Electrochemical Characterization

**Figure 2:**
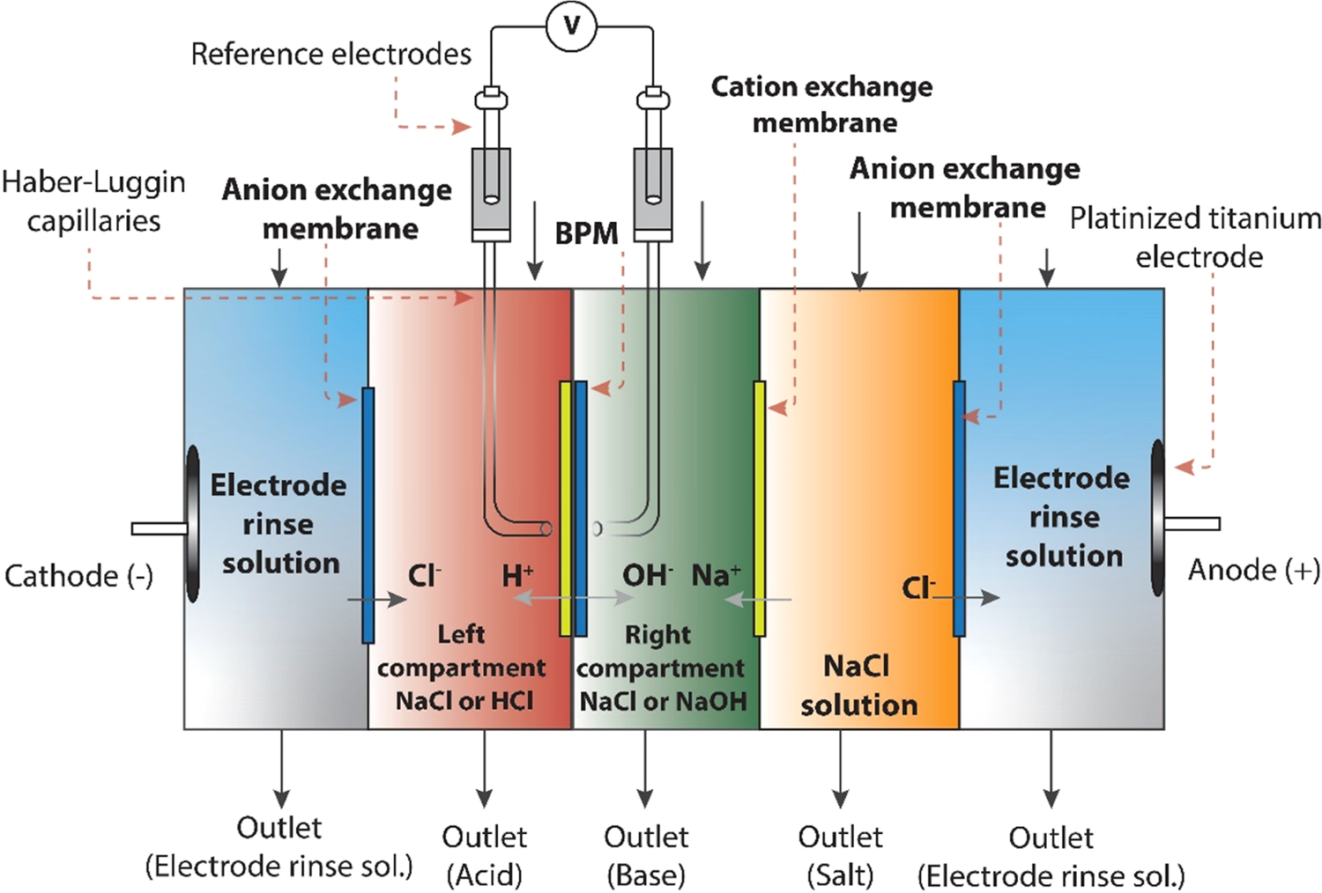
Schematic representation of the five-compartment setup equipped with the Haber–Luggin capillaries with Ag/AgCl reference electrodes for *I*–*V* measurements of the BPMs.

Electrochemical characterization of the bipolar membranes was performed using a homemade PMMA five-compartment testing cell (see Figure 2). Various solution concentrations were used depending on the type of testing. NaCl solutions of 1 and 0.1 M concentrations were used during water dissociation characterization and current efficiency measurement, respectively. Meanwhile, solutions of 0.5 M HCl and 0.5 M NaOH were utilized during water formation operations with BPMs. Each compartment was separated by a different ion-exchange membrane (Fumatech FKB-PK-75/FAB-PK-130) with a total membrane active area of 7 cm^2^ by placing the bipolar membrane in between two plastic plates with circular holes. Furthermore, the setup consisted of two platinized titanium electrodes (Magneto Special Anodes, Schiedam, The Netherlands) placed in the electrode compartments. Two Haber–Luggin capillaries were positioned at both sides of the BPM and connected to two Ag/AgCl reference electrodes (3 M KCl; QM711X, QIS, The Netherlands) to measure the voltage drop across the bipolar membrane. The reference electrodes were connected to a potentiostat (IviumStat.XRi, Ivium Technologies, The Netherlands) to register the voltage drop. The electrolyte solutions of the anode and cathode consisted of 0.25 M iron (II) chloride and 0.25 M iron (III) chloride. All solutions were circulated at a rate of 400 mL/min through the cell compartments using Masterflex pumps. An in-depth investigation of bipolar membranes with impedance spectroscopy will be conducted in a separate study as this requires a rigorous theoretical approach and experimental tests.

### 2.5 Current Efficiency and Energy Consumption

Current efficiency and energy consumption of acid and base production were measured in 0.5 M NaCl solution by recording the pH change of the acid or base compartments. Based on our experience, the pH is more stable in the base compartment and less stable in the acid compartment due to the high mobility of protons (H^+^). The current efficiency and energy consumption were calculated following the equations given below
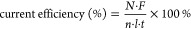
1where *N* is the number of equivalents of hydrochloric acid, *n* is the number of bipolar membranes (*n* = 1 for this system), *F* is the Faraday constant (96485 C/mol), *I* is the current (*A*), and *t* (s) is the duration of the experiment (water dissociation).
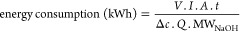
2where *V* is the voltage across the BPM, *I* is the applied current density, *A* is the active area of the BPM, *t* is the process time, *c* is the NaOH concentration change, *Q* is the amount of water recirculated, and MW_NaOH_ is the molecular weight of NaOH (39.99 g/mol). Current efficiency and energy consumption allow a comparison of bipolar membranes in terms of water dissociation (i.e., acid and base generation) at a given current density. To compare the produced BPMs, the generation of acid and base was measured under galvanostatic conditions at current densities of 100 and 400 A/m^2^.

## 3 Results and Discussion

### 3.1 Morphological Characterization of the Fabricated BPMs

**Figure 3:**
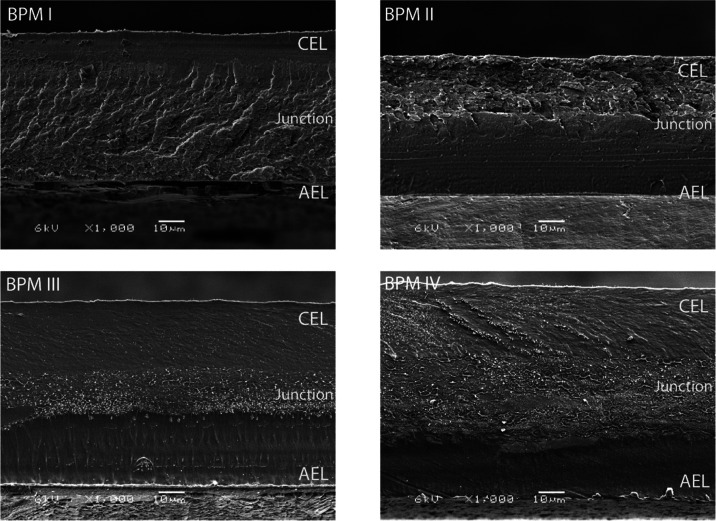
Cross-sectional SEM images of the fabricated BPMs with different total thicknesses and intermediate 3D junctions.

Cross-sectional SEM images of the fabricated BPMs are shown in Figure 3. All images show completely densified layers of the BPM, which are both pore- and crack-free after the process of hot-pressing.

**Figure 4:**
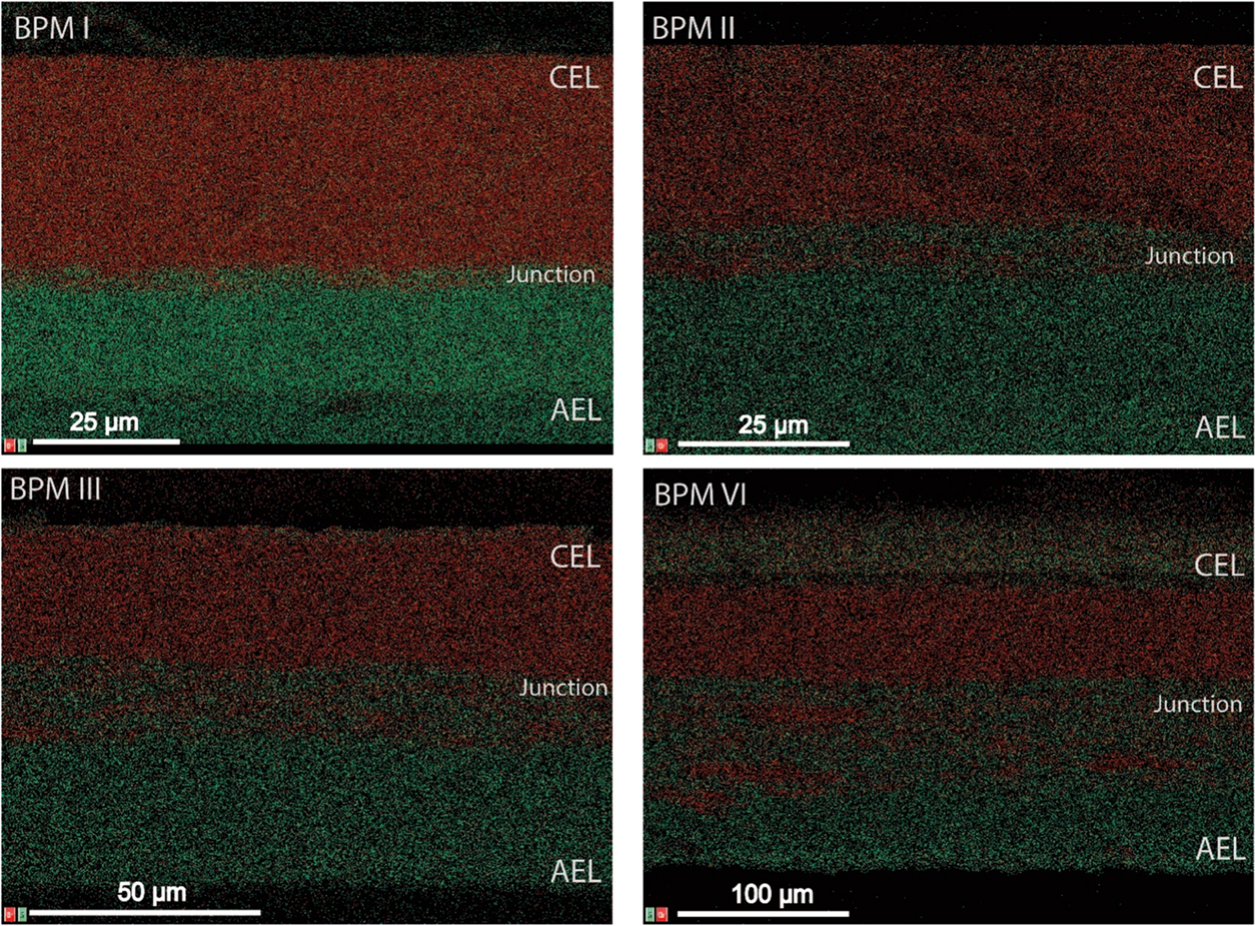
Cross-sectional SEM-EDX images of the fabricated BPMs with different intermediate 3D junctions; elemental mapping for sulfonate (red) and bromide ions (green).

SEM-EDX images, as shown in Figure 4, clearly show the presence of the three different types of layers, as identified by sulfonate ions (in red), which correspond to the cation-exchange polymer, and bromide ions (in green), which correspond to the anion-exchange polymer. We can see the intermediate regions, where both are present, revealing the regions of the cation- and anion-exchange fiber entanglement (i.e., the 3D junction). These entanglement regions (3D junctions) appear with mixed signals of both sulfonate and bromide ions (red and green), and their thicknesses are estimated as listed in Table 3.

**Table 3:** Dry and Wet Thicknesses of the Final BPMs and Thicknesses of the 3D Junctions as Estimated from the EDX Elemental Mapping Analysis

BPM	dry thickness (μm)	wet thickness (μm)	3D junction thickness (μm)
BPM I	65 ± 1	72 ± 2	∼4
BPM II	71 ± 1	78 ± 3	∼8
BPM III	83 ± 1	97 ± 5	∼17
BPM IV	103 ± 1	116 ± 5	∼35

The BET adsorption method was used to collect specific surface area data for different samples of electrospun BPMs and 3D junctions, as presented in Figure S1 of the Supporting Information. The specific surface area is measured for samples containing three layers of the BPM (CEL, 3D junction, and AEL), thus containing two types of nano-/microfibers for both SPEEK and FAA-3/P4VP. The BET specific surface area measurement was conducted after the electrospinning step and before hot-pressing, while the electrospun BPM is still in its porous phase. After the hot-pressing step, the BPM film becomes fully dense. The trend found was a higher specific surface area for an increasing junction thickness. This was confirmed for 3 out of 4 different bipolar membranes formed. The BET surface area of the electrospun fibers of the 3D junction could only be determined experimentally before hot pressing, as hot pressing usually delivers a very dense and transparent bipolar membrane.

FAA-3/P4VP fibers possess larger diameters of 324 ± 55 nm in comparison to SPEEK nanofibers, which have a fiber diameter of 104 ± 18 nm, thus correlating to a lower specific surface area. The two-fiber entanglement at the junction has a high specific surface area of 8.809 m^2^/g. As the only variable among the fabricated BPMs is the thickness of the 3D junction, the thicker the junction, the higher will be the specific surface area of the whole membrane (as shown in Figure S1).

The specific surface areas averaged around 5–6 m^2^/g for all BPMs of different junction thicknesses, while the specific surface area for the 3D junction was measured to be 8.8 m^2^/g. It is worth noting that this specific surface area corresponds to the fibrous structure of the membrane before hot-pressing. We could hypothesize that the interfacial area of contact between CE and AE fibers will deviate drastically from the reported numbers for these specific surface areas. However, it still should represent the best indication of the exact specific surface area of the junction after the hot-pressing step, as measuring or modeling the properties of the microstructural 3D junction is challenging.^22,28^

**Figure 5:**
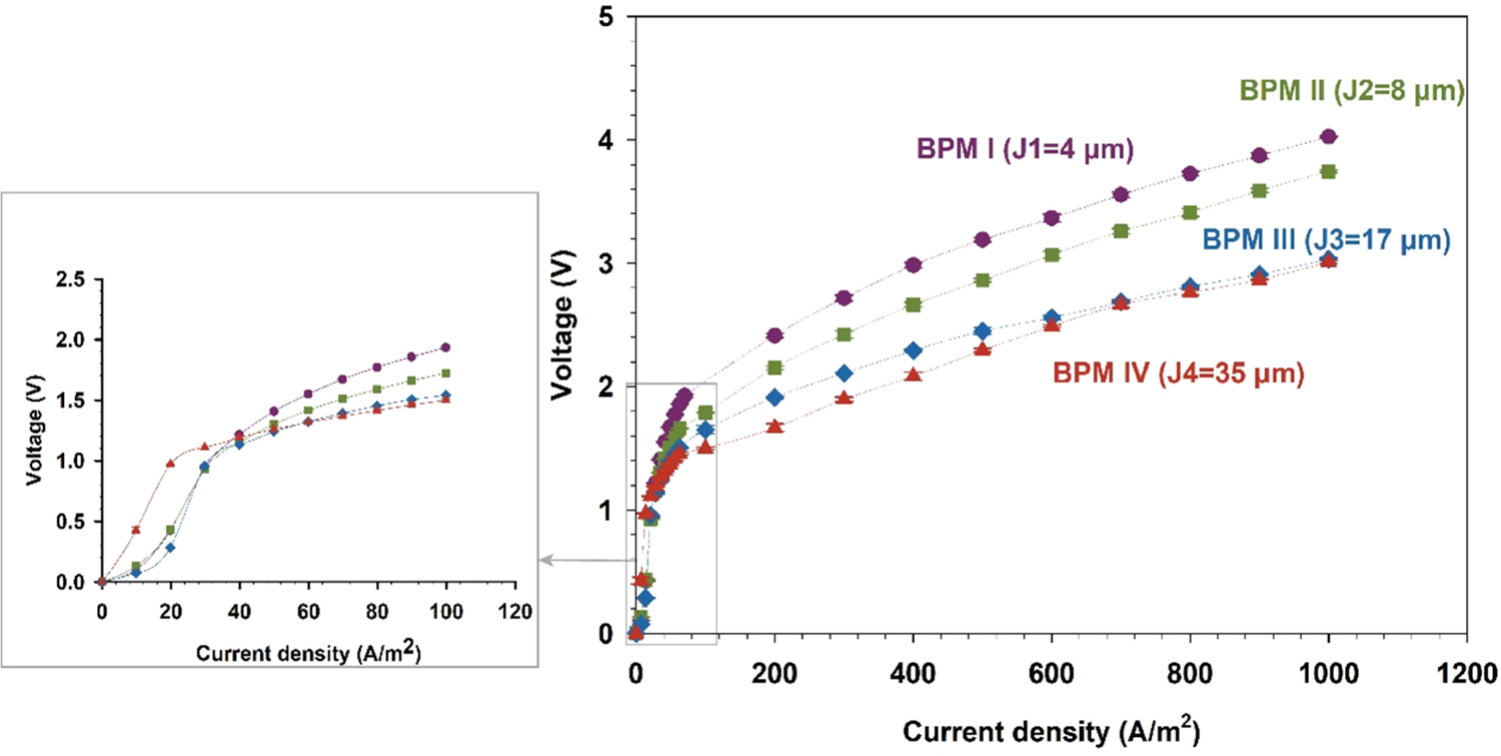
Current–voltage curve of fabricated BPMs under reverse bias (water dissociation) in 1 M NaCl solution. Zoomed out curve: performance of BPMs in the range of 0–100 A/m^2^.

Figure 5 shows the *I*–*V* curve as a measure of the electrochemical performance of the fabricated BPMs in 1 M NaCl solution. Interestingly, water dissociation starts at a current density of ∼20 to 25 A/m^2^ for BPM IV, while for all other BPMs, water dissociation starts at a higher current density (∼35 A/m^2^). This effect can be related to the concept of BPM limiting the current density, a phenomenon attributed to the salt contained within BPM, the selectivity of the BPM toward co-ions (i.e., Na^+^ and Cl^–^), and the bulk concentration.^29^

With increasing junction thickness (i.e., from BPM I to BPM IV), a decrease of the voltage drop across the BPM by 32% is measured when only considering the voltage above the theoretical water dissociation threshold of 0.83 V. Such a decrease of the transmembrane voltage is attributed here to the increase of the interfacial area of the cation/anion material entanglement at the 3D junction as a result of the increase of fiber entanglement at the 3D junctions with varied thicknesses. Clearly, increasing the thickness of the BPM junction has the potential to decrease the overpotential across the BPM, which translates into decreased energy consumption.

The incorporated P4VP polymer acts as a water-dissociation catalyst.^24,30^ It has also been reported to be a carrier polymer, enhancing the “electrospinnability” of other polymers.^31^ The choice to introduce P4VP as a catalyst in the organic medium of the FAA-3 anion-exchange ionomer was based on technical considerations, as the electrospinning equipment LE-50 had only two independent positions for electrospinning. Only with three independent electrospinning positions is a rigorous fabrication of electrospun bipolar membranes feasible because then the anion ionomer solution, the cation ionomer solution, and the catalyst solution can be independently controlled and applied to the junction, for instance. The selection of a polymer that has both good electrospinning and catalytic properties is one of the biggest challenges in the development of electrospun BPMs. In this regard, we investigated several polymeric materials that exhibit potential water-dissociation catalytic activity, although without success. Some of these polymers are poly(vinyl alcohol) (PVA), polyacrylonitrile (PAN), polyacrylic acid (PAA), and electrically conductive polymers such as poly(3,4-ethylene dioxythiophene):polystyrene sulfonate PEDOT:PSS and polypyrrole (PPy).^30,32,33^ Integration of such polymers, however, was not possible because of the relative complexity of the electrospinning process and several experimental factors such as the solubility of the catalytic polymer in the chosen solvent (such as DMAc), the electrospinnability of the polymer, and the blend compatibility between the catalytic and ion-exchange polymers (i.e., FAA-3).

### 3.2 Membrane Performance in Water Formation (Forward Bias)

Although BPMs are mostly used for water dissociation (reverse bias), recent research^34−36^ has focused on the use of BPMs in the opposite mode (i.e., water formation or forward bias). During water formation, protons and hydroxide ions combine to form water in the BPM. Ideally, water formation takes place at (and adjacent to) the junction of the BPM, assuming perfect selectivity of the CEL toward hydroxide ions (OH^–^) and the AEL toward protons (H^+^).

**Figure 6:**
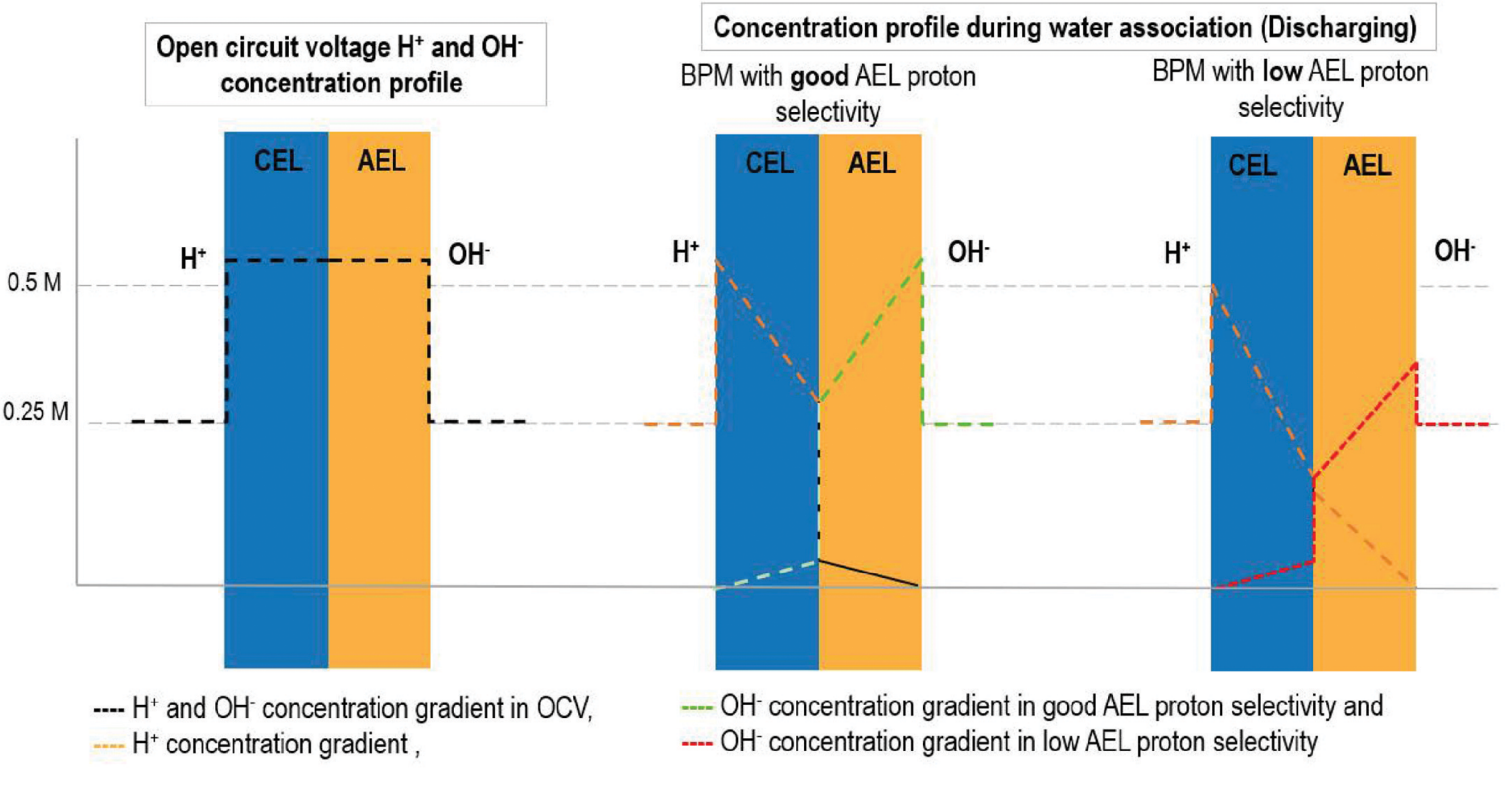
Water formation profiles across the BPM at different conditions.

However, the BPM monopolar layers are less selective, resulting in the undesired transport of hydroxide ions and protons toward the other side, leading to a gradient decline of ion concentration profiles across the BPM, which also means a lower concentration of OH^–^ at the anion-exchange side due to depleted OH^–^ by neutralization with leaked H^+^,^37^ as depicted in Figure 6.

**Figure 7:**
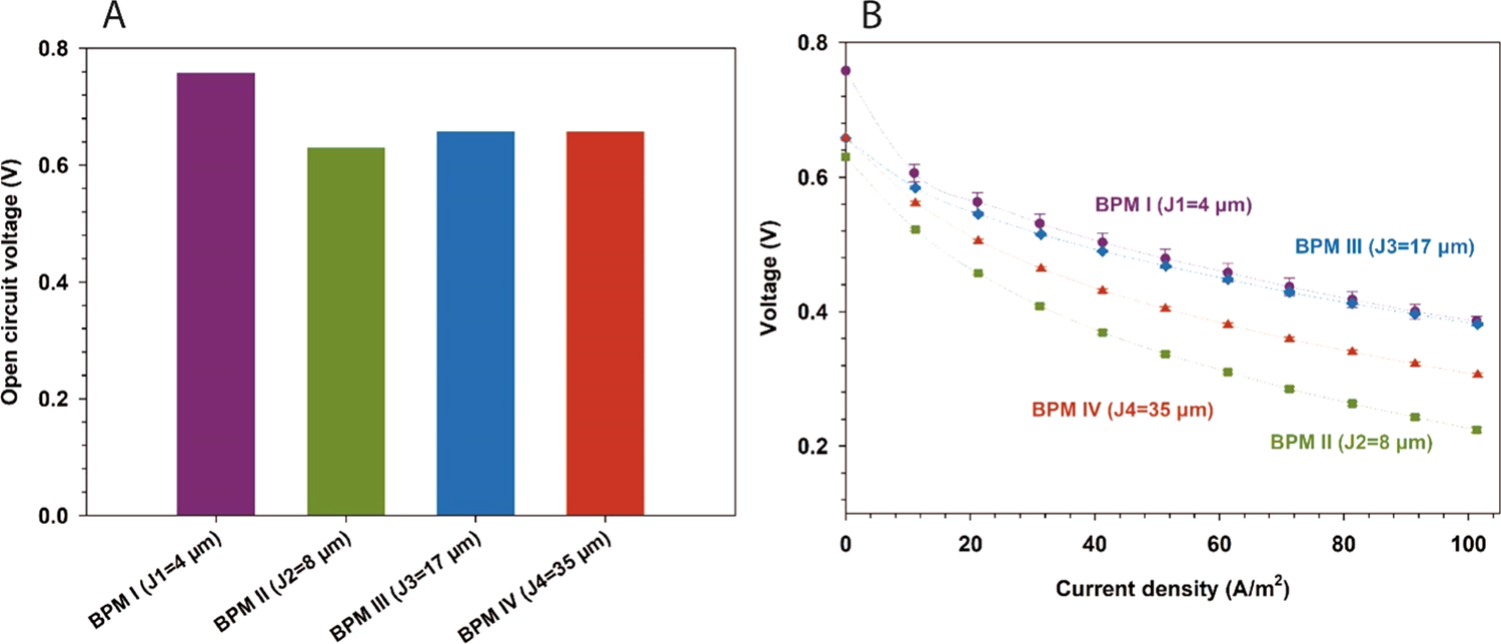
(A) Open-circuit voltage of the fabricated BPMs with different thicknesses. (B) Polarization curves under water formation (forward bias) of the fabricated BPMs in 0.5 M HCl/0.5 M NaOH.

Open-circuit voltages (OCVs) of the fabricated BPMs are presented in Figure 7A, measured using 0.5 M HCl and NaOH (at the CEL and AEL sides of the membrane, respectively). The recorded OCVs ranged between 0.62 and 0.72 V without any major observable difference, except for the higher OCV of BPM I with the thinnest junction (4 μm). Generally, these values are lower than the theoretical value calculated from the Nernst equation (i.e., 0.792 V at 25 °C). All BPMs in this work have the same monopolar layer (AEL and CEL) thicknesses (∼30 μm) and polymer compositions. The AEL (FAA-3/P4VP) permselectivity toward H^+^ is low owing to the decline of BPM OCV and water formation voltage.^38,39^ In addition, the composition of the BPM junction and catalysts contributes toward the OCV and water proton–hydroxide ion combination behavior in the BPM forward bias.^40^ An increase in the thickness of the 3D junction will lead to more contact area between the anion and cation polymers. However, this may be at the expense of more co-ion transport of protons and hydroxyl ions. Such an increased co-ion transport can be detected when exposing the bipolar membrane at OCV to a pure acid (like HCl solution) and a pure base (like NaOH solution).

The performance of the fabricated BPMs during water association was also assessed by applying current in a forward bias mode. In general, BPMs exhibited a relatively sharp drop in the voltage in this range (0–100 A/cm^2^), as shown in Figure 7B. Such a voltage drop across the BPM occurs due to the excessive co-ion transfer, driven mainly by the negative current in the “forward bias mode” applied during water association. It should be noted that the anion-exchange polymers (i.e., FAA-3) used in the BPM fabrication have high ionic conductivity but not sufficient proton-blocking properties.^24,41^ Such a property is crucial for controlling ionic and protonic transport during water association, thus maintaining a lower voltage drop.

### 3.3 Current Efficiency under Water Dissociation

Current efficiency is determined from the amount of HCl/NaOH generated at a certain current density, and it has been measured for the fabricated BPMs in 0.1 M NaCl solution at different current densities of 100 and 400 A/m^2^. The current efficiency is evaluated by measuring the acid (HCl) and base (NaOH) generation by the pH change in the acid and base compartments and comparing that to the theoretical values as calculated from eq 1.

**Figure 8:**
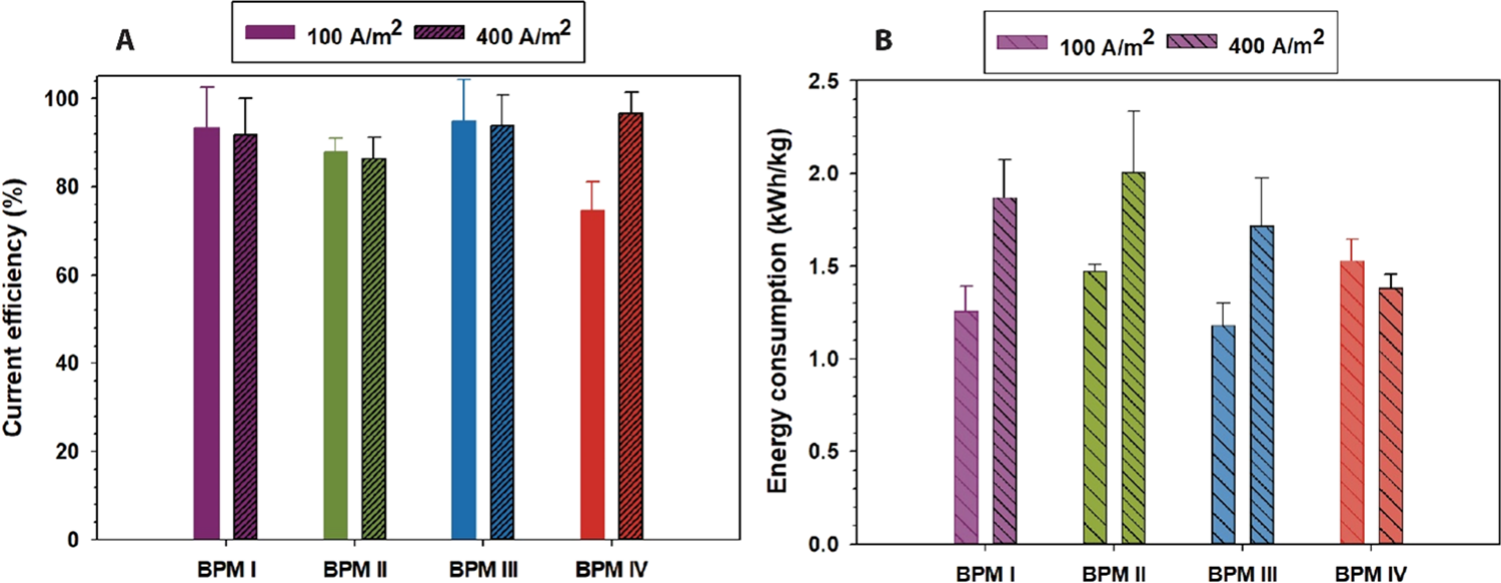
(A) Current efficiency of the fabricated BPMs in 0.1 M NaCl at current densities of 100 and 400 A/m^2^. (B) Energy consumption in kWh/kg for generating 1 kg of equivalent NaOH using the fabricated BPMs at both current densities of 100 and 400 A/m^2^.

High current densities and low electrolyte concentrations are optimal for BPM operation,^36^ as illustrated in Figure 8, where the measured efficiencies had average values greater than 90%. Figure 8 shows that there is no statistically significant relationship between the thickness of the BPM 3D junction and the current efficiency during water dissociation. We could observe that the current efficiency of BPM IV at 100 A/m^2^ is abnormally and inexplicably lower than the average current efficiency of all BPMs at both current densities of 100 and 400 A/m^2^. Energy consumptions (estimated from eq 2) for BPMs with thicker 3D junctions, which run at lower voltages, are 1.2–2 kWh/kg (Figure 8B), as almost all BPMs have relatively high current efficiencies.

## 4 Conclusions

Several bipolar membranes (BPMs) with different 3D junction thicknesses have been successfully fabricated with electrospinning by varying the time required to deposit the nano-/microfibers at the junction. This study included the fabrication of four BPMs with 3D junction thicknesses of approximately 4, 8, 17, and 35 μm to evaluate the effect of the junction thickness on the performance of the electrospun hot-pressed BPMs.

Water dissociation curves of BPMs showed a lower voltage for the BPMs with a larger thickness as they are associated with the three-dimensional increase of the interfacial contact area between the cation- and anion-exchange fibers, hence increasing the effective reaction area for water dissociation. Increasing the BPM thickness from 4 μm (BPM I) to 35 μm (BPM IV) resulted in a decrease of the BPM water dissociation overpotential by 32%.

The evaluation of current efficiency indicated efficient production of acid (HCl) and base (NaOH) with a high efficiency ranging between 90 and 100%. Furthermore, a comparison of BPM performance during the water association operation showed a sharp drop in the voltage from the levels of its given open-circuit voltage (OCV) due to high hydroxide ion (OH^–^) and proton (H^+^) leakage through the corresponding layers. For BPMs to operate effectively in both modes (water dissociation and association), the development of BPMs with highly selective anion- and cation-exchange layers is therefore needed.

## Supporting Information Available

Specific surface area as measured by the BET of the electrospun materials of BPMs for different junction thicknesses and only the 3D junction (Figure S1) (PDF)
