# Supplementary material for: Understanding the Impact of the Three-Dimensional Junction Thickness of Electrospun Bipolar Membranes on Electrochemical Performance
Source: ACS Appl Polym Mater. 2023 Apr 4;5(4):2533–41. doi: 10.1021/acsapm.2c02182 (PMC10112390; doi:10.1021/acsapm.2c02182)
Supplement: Supplementary file 1 — ap2c02182_si_001.pdf [file ap2c02182_si_001.pdf]

Supporting information

Understanding the impact of three-dimensional junction thickness of electrospun bipolar membranes on electrochemical performance

Emad Al-Dhubhani a,c, Jan W. Post a, Marat Duisembiyev b, Michele Tedesco a, Michel Saakes a*

a Wetsus, European Centre of Excellence for Sustainable Water Technology, Oostergoweg 9, 8911 MA Leeuwarden, The Netherlands

b L.N. Gumilyov Eurasian National University , Satpayev str. 2, 010008 Astana, Repulic of Kazakhstan

c Membrane Science and Technology, University of Twente, P.O. Box 217, 7500 AE, Enschede, The Netherlands

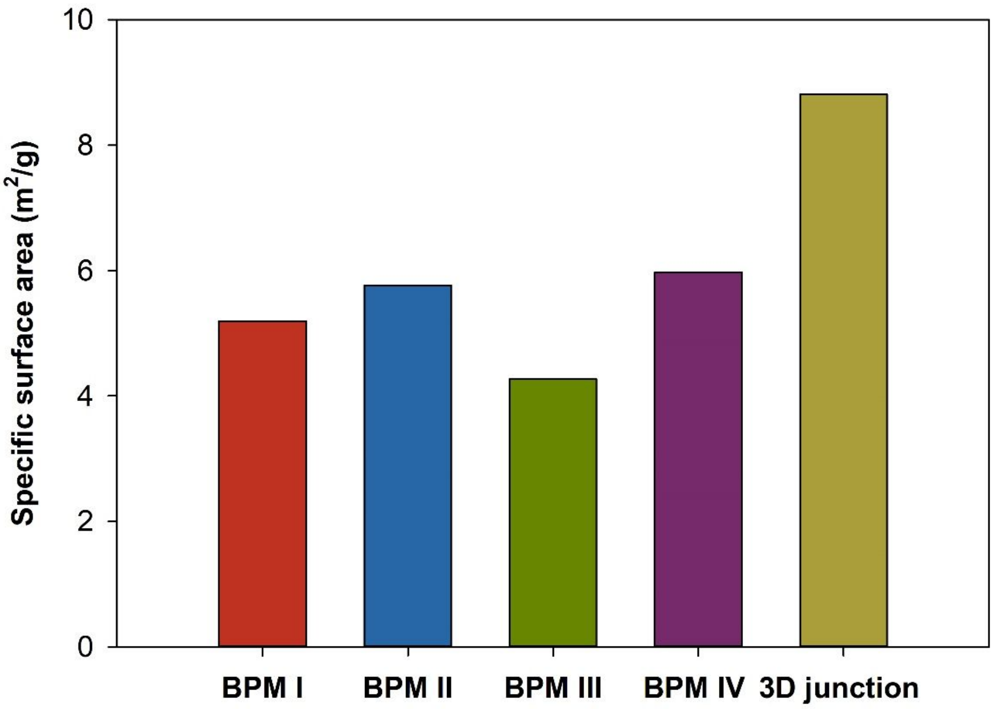

Figure S1: Specific surface area as measured by BET of the electrospun materials of BPMs for different junction thickness and only 3D junction:
